# Vasovagal tonus index (VVTI) as an indirect assessment of remission status in canine multicentric lymphoma undergoing multi-drug chemotherapy

**DOI:** 10.1007/s11259-017-9695-8

**Published:** 2017-08-08

**Authors:** Evi Pecceu, Brittainy Stebbing, Yolanda Martinez Pereira, Ian Handel, Geoff Culshaw, Hannah Hodgkiss-Geere, Jessica Lawrence

**Affiliations:** 1 Royal (Dick) School of Veterinary Studies & Roslin Institute, University of Edinburgh, Roslin, EH25 9RG UK; 2 Present Address: Small Animal Teaching Hospital, University of Liverpool, Liverpool, CH64 7TE UK; 3 Present Address: College of Veterinary Medicine, University of Minnesota, St Paul, MN 55108 USA

**Keywords:** Lymphoma, Heart rate, Chemotherapy, Remission, Electrocardiogram

## Abstract

Vasovagal tonus index (VVTI) is an indirect measure of heart rate variability and may serve as a marker of disease severity. Higher heart rate variability has predicted lower tumour burden and improved survival in humans with various tumour types. The purpose of this pilot study was to evaluate VVTI as a biomarker of remission status in canine lymphoma. The primary hypothesis was that VVTI would be increased in dogs in remission compared to dogs out of remission. Twenty-seven dogs were prospectively enrolled if they had a diagnosis of intermediate to high-grade lymphoma and underwent multidrug chemotherapy. Serial electrocardiogram data were collected under standard conditions and relationships between VVTI, remission status and other clinical variables were evaluated. VVTI from dogs in remission (partial or complete) did not differ from dogs with fulminant lymphoma (naive or at time of relapse). Dogs in partial remission had higher VVTI than dogs in complete remission (*p* = 0.021). Higher baseline VVTI was associated with higher subsequent scores (*p* < 0.001). VVTI also correlated with anxiety level (*p* = 0.03). Based on this pilot study, VVTI did not hold any obvious promise as a useful clinical biomarker of remission status. Further investigation may better elucidate the clinical and prognostic utility of VVTI in dogs with lymphoma.

## Introduction

Heart rate variability (HRV) is the physiologic variation in the beat-to-beat interval, or the R-R interval on an electrocardiogram (ECG) recording, which correlates with cardiac autonomic tone (Kuo et al. 2005). Variations in R-R intervals reflect vagal outflow as parasympathetic nerve traffic exerts its effects much faster than sympathetic outflow (Task Force 1996). In people, HRV is an index of cardiovascular and mortality risk in health and cardiac and renal disease (Tsuji et al. 1996; Kiviniemi et al. 2007; Oikawa et al. 2009). Several studies have investigated HRV in healthy and unhealthy dogs, with diseases including myocarditis, myxomatous mitral valve disease and diabetes mellitus, suggesting a role for VVTI as a predictive or prognostic biomarker (Calvert and Wall 2002; Doxey and Boswood 2004; Pereira et al. 2008; Manzo et al. 2009; Oliveira et al. 2012; Pirintr et al. 2012; Rasmussen et al. 2012; López-Alvarez et al. 2014; Martlé et al. 2014; Bogucki and Noszczyk-Nowak 2015).

The vagus nerve may be intricately involved in human cancer as part of an inflammatory reflex, in which tumour-mediated inflammation triggers vagal afferent traffic to the brain to modulate immune and neuroendocrine function (Tracey 2002; Gidron et al. 2005; Mravec et al. 2006; Gidron and Ronson 2008; Golan et al. 2009; Rosas-Ballina and Tracey 2009; Irwin and Cole 2011; Olofsson et al. 2012). Recent studies have investigated the prognostic value of HRV in human cancer patients. Higher HRV was associated with longer survival time, and predicted lower tumour burden and improved survival in patients with various solid tumours (Dekker et al. 1997, Hoffmann et al. 2001, Chiang et al. 2010, Fadul et al. 2009, Giese-Davis et al. 2015, Guo et al. 2015, Kim et al. 2015). Thus HRV has potential in oncology as a biomarker of response to chemotherapy and overall prognosis (De Couck and Gidron 2013; Giese-Davis et al. 2015).

Vasovagal tonus index (VVTI) is a time domain indicator of HRV that quantifies high-frequency variations in heart rate. VVTI measurement is rapid, non-invasive and uncomplicated because it can be calculated from ECG recordings using a simple mathematical formula. Its prognostic value has been demonstrated in dogs with congestive heart failure secondary to both dilated cardiomyopathy and myxomatous mitral valve disease (Häggström et al. 1996; Doxey and Boswood 2004; Pereira et al. 2008; López-Alvarez et al. 2014). If VVTI is a reliable biomarker of disease severity, it may therefore have widespread clinical potential. Recognizing that humans with higher disease burden have significantly lower HRV compared to patients with earlier cancer stages (Entschladen et al. 2004; Gidron et al. 2005; Mantovani et al. 2008; De Couck et al. 2012; De Couck and Gidron 2013), we elected to evaluate the ability of VVTI to detect tumour burden in dogs with lymphoma. Lymphoma burden decreases rapidly in most dogs following chemotherapy, thereby permitting VVTI comparisons within each dog and across groups of dogs. The primary hypothesis of this study was that vagal tone, and therefore HRV, would increase in dogs with intermediate to high-grade lymphoma when in remission with multidrug chemotherapy.

## Material and methods

### Dogs

Client-owned dogs with multicentric non-Hodgkin’s lymphoma were prospectively enrolled at a specialty oncology service between January 2014 and September 2015. Inclusion criteria were: stage III-V intermediate and high grade lymphoma diagnosed by cytology or histology, treatment with standardised multidrug (cyclophosphamide, hydroxydaunorubicin, vincristine, prednisone or CHOP) chemotherapy and client consent (Macdonald et al. 2005). As this was a pilot study, dogs were enrolled at any stage of treatment or remission, including at diagnosis, while undergoing chemotherapy or during routine monthly monitoring for relapse.

Exclusion criteria included evidence of behavioural aggression, systemic diseases or obvious pre-existing cardiac disease identified from a combination of clinical history, physical examination and/or echocardiography. Pre-chemotherapy echocardiography was at the discretion of the clinician, and principally performed to identify subclinical cardiac disease prior to administration of doxorubicin. The study was approved by the Institutional Veterinary Ethics and Review Committee.

### ECG recordings and VVTI measurements

Standard 6-lead ECGs were recorded and printed (50 mm/s) with a multi-channel ECG machine (Schiller AT-102 Plus™) collected at every chemotherapy or follow-up visit. All recordings were obtained from unsedated dogs placed in right lateral recumbency in a dimly-lit room at least 2 h following admission. ECG leads were attached with atraumatic clips and alcohol (95% ethanol, 5% methanol; Surgical Spirit™, Vet Way Ltd., Elvington York, UK) and no electronic filters were applied. ECG trace analysis was performed manually on the first available 20 consecutive R-R intervals of sinus origin. Each dog was scored during ECG acquisition according to a 5-point rising scale of anxiety (Table 1). This anxiety score was generated by the authors using elements from anxiety scores previously published (Beata et al. 2007; Frank et al. 2006). R-R intervals were measured in millimetres and converted into milliseconds. VVTI was calculated as the natural logarithm of the variance of 20 consecutive R-R intervals as previously described (Häggström et al. 1996). This was performed by one member of the Cardiology Service (BS) who was blinded to the clinical information. Follow-up ECGs were scheduled at monthly intervals for the first 18 months, followed by every other month for 18 months.

### Clinical data

Signalment was collected for each dog as well as clinical data where available, including the presence or absence of hypercalcaemia, stage, substage, immunophenotype, chemotherapy drugs and dosages administered, remission status, duration to remission, duration to first relapse, and development of chemotherapy-induced toxicity. Full staging was recommended for all dogs at diagnosis, however not always pursued according to clients’ wishes. Minimal staging, which included lymph node measurements, haematology and biochemistry, was performed in all dogs prior to initiation of chemotherapy. Remission status was determined using standardised criteria (Vail et al. 2010). Dogs with overt lymphoma at the time of initial diagnosis or at time of relapse were documented as “out of remission (OR)” while remission status was divided as partial remission (PR) or complete clinical remission (CR). At each chemotherapy visit, lymph node measurements, physical exam and haematology was performed. Chemotherapy-induced toxicity was recorded using standardised Veterinary Cooperative Oncology Group (VCOG) criteria (VCOG 2011). At time of relapse, repeat CHOP was offered. For dogs that failed CHOP chemotherapy (relapsed disease during CHOP), lomustine and prednisolone was offered as first-line rescue therapy.

### Statistical analysis

Statistical analyses were performed (IH) using the R Statistical System (R Core Team. R: A language and environment for statistical computing. R Foundation for Statistical Computing (R Core Team 2015) . Available at: https://www.R-project.org/.). VVTI scores were compared using linear models (estimated with lme function of the R nlme package) (Pinheiro et al. 2017). Case identifier was included as a random effect on the model intercept to reduce the impact of pseudo-replication when measurements were repeated within dogs. A first order autocorrelation term was included as VVTI measurements demonstrated autocorrelation on exploratory analysis (using the AR1 function of the R nlme package). The significance of model terms was assessed using likelihood ratio tests (LRT) on the addition of the terms. Models assessing impact of remission status used categorical remission status as a single fixed effect. Remission status was retained as a fixed effect in models assessing anxiety, substage and age. The association between baseline VVTI and later VVTI was described by Pearson product moment correlation coefficient and estimated with a random effects linear model. Predictability of remission by means of a VVTI was evaluated with a binary logistic regression model. The study involved multiple statistical tests in the exploration of hypotheses. No explicit corrections were made for this; *p*-values are presented as-is. Data are expressed as mean, median and range. A *p*-value of <0.05 was considered statistically significant for reporting of final variables from the models.

## Results

**Fig. 1:**
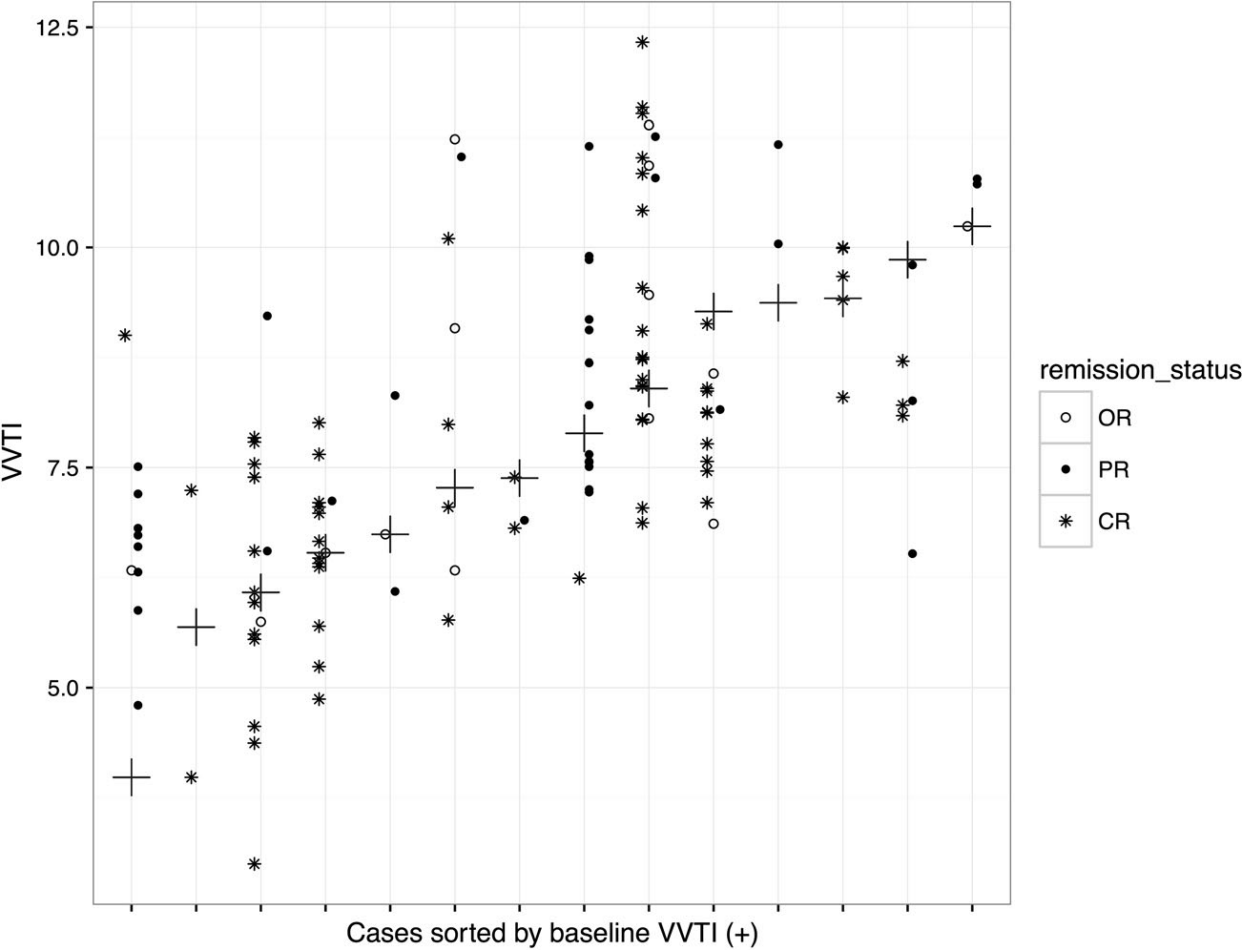
Vasovagal tonus index (VVTI) values per dog in different remission statuses for dogs with available baseline scores at time of initial diagnosis (out of remission = OR) and subsequent partial remission (PR) and/or complete remission (CR) values (*N* = 14). Baseline VVTI is represented by a +

Twenty-seven dogs with a diagnosis consistent with non-Hodgkin’s lymphoma were enrolled in the study. Demographics and clinical data for all dogs are shown in Table 2. Twelve dogs had echocardiography prior to doxorubicin administration. A total of 249 VVTIs were obtained, with a mean and median of 9 scores/dog and 7 scores/dog (range of 1–31 scores/ dog). Sixteen dogs (59%) had a baseline VVTI at initial diagnosis (chemotherapy-naïve disease) while the remaining dogs were enrolled following initiation of chemotherapy. Of the 16 dogs with baseline VVTI scores,14 had serial VVTI scores while in PR or CR; 2 dogs did not achieve remission (Fig. 1). Of the 11 dogs lacking baseline VVTI data prior to initiation of chemotherapy, four dogs relapsed over time, providing a VVTI when OR at least once.

**Table 2:** Summary of clinical data from 27 dogs with lymphoma in which vasovagal tonus index (VVTI) was measured

Clinical Characteristics (*N* = 27 dogs)		
Age (years)	Median (Range)	8 (2.25–11.91)
Sex	M	18 (67%)
	F	9 (33%)
Body weight (kg)	Median (Range)	26.6 (6.3–75.5)
Breed	Labrador	4 (15%)
	Border Collie	3 (11%)
	Boxer	3 (11%)
	Rottweiler	2 (7.5%)
	WHWT	2 (7.5%)
	Cross	3 (11%)
	Other (<2)	10 (37%)
Breed type	Brachycephalic	3 (11%)
Stage	Stage III	9 (33%)
	Stage IV	15 (56%)
	Stage V	3 (11%)
Substage	Substage a	19 (70%)
	Substage b	8 (30%)
Hypercalcemia		5 (19%)
Immunophenotype	B-cell	4 (80% of samples tested)
	T-cell	1 (20%)
VVTI total scores	242	38 OR
		61 PR
		143 CR
VVTI measures	OR	(mean 7.77 median 7.48 IQ 6.58–9.08)
	PR	(mean 8.00 median 8.12 IQ 6.81–8.93)
	CR	(mean 7.54 median 7.54 IQ 6.55–8.33)

*WHWT* West Highland White Terrier, *OR* out of remission, *PR* partial remission, *CR* complete remission, *IQ* Interquartile range

**Table 1:** Anxiety scoring

Anxiety score	0 = no; 1 = yes
Attempt to rise	
Stiff / tense	
Panting	
Limb withdrawal	
Awake	
Overall score /5 =

**Fig. 2:**
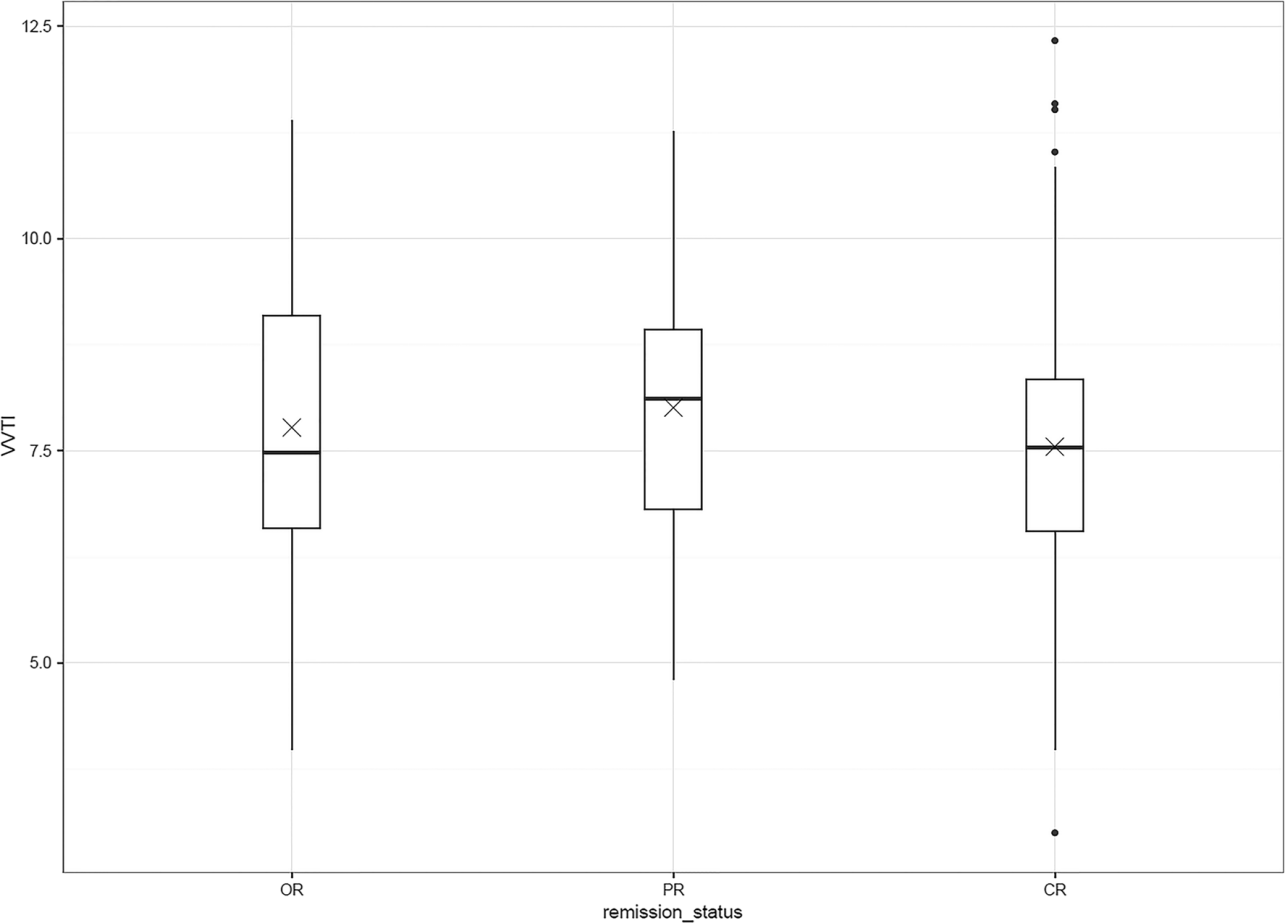
Box and Whisker plot demonstrating the distribution of vasovagal tonus index (VVTI) values across remission status for the 27 dogs enrolled; horizontal line represents the median, the X represents the mean of scores. VVTI was significantly higher in dogs in partial remission (PR) compared to dogs in complete remission (CR). VVTI from dogs with fulminant lymphoma either at initial diagnosis or at time of relapse (out of remission = OR) were not significantly different from dogs in partial and/or complete remission

No significant differences were detected between VVTIs from dogs that were OR (median 7.48, range 3.98–11.39, *n* = 38) compared to dogs in either PR or CR (Fig. 2). Compared to CR tracings (median 7.54, range 3.90–12.33, *n* = 143), VVTI was higher in PR tracings (median 8.12, range 4.8–11.26, *n* = 61) with a mean difference of 0.59 (*p* = 0.021) (results with interquartile ranges in Table 1). Seven scores were obtained from dogs with stable disease (SD) or progressive disease (PD) and these scores were not included in analysis.

Dogs with higher baseline scores were more likely to have higher subsequent scores when in partial or complete remission (Fig. 1); for every unit increase in baseline VVTI, there was an average 0.62 increase in VVTI (*p* < 0.001). VVTI was associated with anxiety level, with decreasing VVTI as anxiety increased (*p* = 0.03). VVTI did not differ between dogs with substage a and b lymphoma (*p* = 0.28). Likewise, VVTI did not correlate with sex, age, lymphoma stage, hypercalcemia, chemotherapy-induced adverse effects, chemotherapy status (receiving or not receiving concurrent chemotherapy, number of treatments, number of doxorubicin treatments), breed or brachycephalic phenotype.

## Discussion

This is the first study to investigate the use of VVTI in canine cancer. Humans with more advanced cancer, have been shown to have significantly lower HRV compared to patients with earlier cancer stages (Entschladen et al. 2004; Gidron et al. 2005; Mantovani et al. 2008; De Couck et al. 2012; De Couck and Gidron 2013). As disease burden in canine lymphoma decreases rapidly following the initiation of chemotherapy, canine lymphoma was a reasonable initial model disease in which to evaluate VVTI, thereby permitting comparisons within each dog and across groups of dogs.

While VVTI was significantly higher for dogs in PR compared to in CR, VVTI in either of these stages did not differ from OR. Therefore the null hypothesis was correct. The results were unexpected, as theoretically, decreased lymphoma burden in dogs in CR compared to tumour burden in PR would lead to decreased sympathetic tone, increased parasympathetic tone and thus increased VVTI. Similarly, it was expected that dogs in either CR or PR would have increased VVTI compared to dogs that were OR. Although it is unclear how HRV directly correlates to human cancer, a bi-directional relationship has been hypothesised. Higher disease burden was thought to decrease heart rate variability due to inflammation, oxidative stress and sympathetic activation. Vagal tone however is not only a consequence of tumour burden but it is also suspected to actively modulate tumour growth through anti-inflammatory effects via two separate ways. Via afferent vagal nerve fibres, the hypothalamic-pituitary-adrenal axis can be activated, leading to the production of corticosteroids and an anti-inflammatory response. Via efferent fibres, anti-inflammatory signals can be transmitted via acetylcholine to activate receptors on tissue macrophages and suppress cytokine synthesis (Gidron et al. 2005; Mravec et al. 2006). Prior studies have shown that there is a worse prognosis in cancer when there is not an intact vagus; moreover, a causal relationship was shown between vagal nerve activation and reduced tumour volume (Erin et al. 2004; Erin et al. 2008; Erin et al. 2012). This bi-directional relationship between cancer burden and heart rate variability hampers a simplistic interpretation of higher tumour burden that results in a lower HRV and thus may offer an explanation for the unexpected results.

Dogs with higher baseline values were found to have higher subsequent VVTI scores when in remission. This suggests that there may be baseline variation across dogs regardless of intra-dog score variability. Previously a range of scores with marked variability across breeds of dogs has been published (Doxey and Boswood 2004). However, in this study, it is not the absolute value of VVTI that is important but rather the change from baseline. Brachycephalic dogs have higher vagal tone and higher VVTI, and while brachcephaly may have affected the degree to which VVTI changed from baseline, only three dogs were brachycephalic, making it unlikely that this significantly impacted the chance to detect a correlation between VVTI scores and remission status. Statistical analysis did not show any significant correlation between VVTI score and brachycephalic phenotype.

Higher anxiety scores were associated with lower VVTI. The protocol to obtain ECG was designed to minimise anxiety during collection of data, however some dogs still demonstrated signs of anxiety and further modifications such as prolonged acclimatisation or permitting owner presence in future studies may further reduce anxiety. As the change in VVTI from baseline was important in this study and anxiety scores did not increase in each dog over time the fact that VVTI scores from dogs in PR was higher than dogs in CR suggests the level of anxiety did not significantly influence our findings.

Dogs in which concurrent cardiac disease was suspected were not included. Echocardiography was not performed in all dogs prior to the first dose of doxorubicin. It is possible that dogs with subclinical cardiac disease were included in this study. While inclusion criteria could have required an echocardiogram prior to treatment, published literature in dogs with mitral valve disease suggests that subclinical cardiac disease would not have significantly affected VVTI (Häggström et al. 1996). Moreover, echocardiogram cannot predict doxorubicin-induced cardiotoxicity in dogs, calling into question its necessity prior to treatment (Ratterree et al. 2012; Tater et al. 2012).

Fewer values were obtained of dogs with fulminant lymphoma compared to dogs in PR or CR for multiple reasons. As canine non-Hodgkin’s lymphoma is rapidly proliferative, rapid remission is achieved in most dogs within one to three weeks of starting chemotherapy. Therefore, dogs contributed more scores when in PR or CR compared to when OR. This study was designed to include dogs already receiving chemotherapy and therefore without baseline values. This was deliberate in order to maximize the number of dogs recruited to the study and to increase the number of samples for analysis. It also ensured that the chemotherapy was initiated at time of diagnosis without delay in treatment to obtain baseline VVTI scores, as sedation was often used for diagnostics.

The impact of chemotherapy on VVTI is unclear although in human oncology, reduced HRV was found following treatment with anthracyclines (Tjeerdsma et al. 1999). Doxorubicin treatments as part of the CHOP protocol in our study may have influenced the VVTI scores in this population, however exploratory analysis did not show any obvious correlation of VVTI with increasing numbers of doxorubicin doses or chemotherapy treatments overall. Additionally, VVTI values from dogs on chemotherapy were not markedly different from dogs off chemotherapy following completion of their protocol. It was not the primary aim of this pilot study to accurately evaluate associations between VVTI and clinical variables other than remission status, however it was interesting to evaluate for hypothesis-generation purposes. Likewise, as the study involved multiple statistical comparisons there was a risk of increased type I errors. There were no clear associations with multiple clinical variables and VVTI, and although it is possible that a larger study could reveal an association, VVTI may instead be an independent variable.

## Conclusions

In conclusion, there were no significant differences in VVTI between dogs in remission compared to dogs with overt lymphoma in this initial pilot study. Surprisingly, PR VVTIs were higher than VVTIs of dogs in CR. This is the first study to indicate that VVTI varies over the course of canine lymphoma treatment. Further studies will be essential to improve understanding of the pathophysiology of autonomic tone in dogs with lymphoma or other neoplastic diseases. A larger study population will be required to determine if clear prognostic potential exists, as has been shown in humans.
